# Hyperintense putaminal rim at 1.5 T: prevalence in normal subjects and distinguishing features from multiple system atrophy

**DOI:** 10.1186/1471-2377-12-39

**Published:** 2012-06-18

**Authors:** Khin K Tha, Satoshi Terae, Akiko Tsukahara, Hiroyuki Soma, Ryo Morita, Ichiro Yabe, Yoichi M Ito, Hidenao Sasaki, Hiroki Shirato

**Affiliations:** 1 Department of Radiobiology and Medical Engineering, Hokkaido University Graduate School of Medicine, N-15, W-7, Kita-ku, Sapporo, 060-8638, Japan; 2 Department of Diagnostic Radiology, Hokkaido University Graduate School of Medicine, N-15, W-7, Kita-ku, Sapporo, 060-8638, Japan; 3 Department of Neurology, Hokkaido University Graduate School of Medicine, N-15, W-7, Kita-ku, Sapporo, 060-8638, Japan; 4 Department of Clinical Trial Management, Hokkaido University Graduate School of Medicine, N-15, W-7, Kita-ku, Sapporo, 060-8638, Japan

## Abstract

**Background:**

Hyperintense putaminal rim (HPR) is an important magnetic resonance imaging (MRI) sign for multiple system atrophy (MSA). Recent studies have suggested that it can also be observed in normal subjects at 3 T. Whether it can be observed in normal subjects at 1.5 T is not known. This study aimed to determine whether HPR could be observed in normal subjects at 1.5 T; and if so, to establish its prevalence, the MRI characteristics, and the features which distinguish from HPR in MSA patients.

**Methods:**

Axial T2-weighted images of 130 normal subjects were evaluated for the prevalence of HPR, its age and gender distribution, laterality, maximum dimension, association with hypointensity of nearby putamen, and presence of discontinuity. To distinguish from that observed in MSA, axial T2-weighted images of 6 MSA patients with predominant parkinsonism (MSA-P) and 15 MSA patients with predominant cerebellar symptoms (MSA-C) were also evaluated. The characteristics of HPR were compared between these patients and age-matched normal subjects. The mean diffusivity (MD) values of putamen were also compared. Fisher’s exact test, *t*-test, and one way analysis of variance were used to determine significance at corrected p < 0.05.

**Results:**

HPR was observed in 38.5% of normal subjects. Age and gender predilection and laterality were not observed. In most cases, it occupied the full length or anterior half of the lateral margin of putamen, and was continuous throughout its length. Maximum transverse dimension was 2 mm. There was no association with hypointensity of nearby putamen. However, in MSA-P, HPR was located predominantly at the posterolateral aspect of putamen, and associated with putaminal atrophy. Discontinuity of HPR was more frequently observed in MSA-P. On visual analysis, the characteristics of HPR were similar between MSA-C patients and normal subjects. Patients with MSA of either type had significantly higher MD values of putamen than normal subjects.

**Conclusions:**

HPR can be observed in 38.5% of normal subjects at 1.5 T. Thin linear hyperintensity without discontinuity, occupying the full length or anterior half of the lateral margin of the putamen, is suggestive of “normal.” In doubtful cases, measurement of the MD values of nearby putamen may be valuable.

## Background

The term “hyperintense putaminal rim (HPR)” is commonly used to represent a linear hyperintensity at the lateral margin of the putamen on T2-weighted or proton-density-weighted images of the brain [1-10]. It was first reported by Savoiardo et al. as an observation in multiple system atrophy (MSA) patients with extrapyramidal symptoms and autonomic failure [1]. Later studies suggested an association of HPR with bradykinesia and contralateral rigidity [2,3]. The clinical applicability of HPR in distinguishing between MSA and Parkinson’s disease (PD) has also been reported [2,6]. That is, because HPR is observed in MSA, but not in PD, it can be used to distinguish between these two diseases having similar clinical features [2,6].

Although HPR is widely accepted as a magnetic resonance imaging (MRI) sign suggestive of MSA, consensus is lacking among the previous studies in regard to whether it is pathological [1-8]. While the mere presence of linear hyperintensity at the lateral margin of the putamen had been considered pathological in many studies [2,6-8], a few studies have suggested that mild linear hyperintensity is a finding of normal aging [4,5]. In the latter studies, HPR was considered as abnormal only if it was moderate to severe in degree and exhibited posterolateral predominance [4], or if it was associated with hypointensity of the nearby putamen [5]. There has been large variation in the reported sensitivity and specificity of HPR for the diagnosis of MSA [4,6-8]. Although slight variation in the scan parameters can influence its appearance, it is considered that this variation is generally attributed to a lack of consensus about HPR. Although there have been suggestions that mild linear hyperintensity may be a normal finding, there is still a lack of statistically significant datum to support this conclusion [4,5].

In 2005, Lee et al. reported that HPR was observed in normal subjects at 3 T, but found that it was not observable in normal subjects at 1.5 T [9]. Two years later, Fujii et al. examined HPR in a large number of subjects, and found that HPR was prevalent in normal subjects between 30 and 70 years of age, at 3 T [10]. Based on their observations, Fujii et al. considered HPR in normal subjects as a “pseudosign” — a perceived hyperintensity due to hypointensity of the remainder of the putamen induced by age-related iron deposition. They also commented that HPR was clearly visible at 3 T, but not at 1.5 T, due to pronounced susceptibility effects at 3 T. In light of the findings of these reports, a few recent studies performed using 3 T have taken into account the possibility of observing HPR in normal subjects [11,12].

Although there is general consensus that HPR is sometimes observed in normal subjects at 3 T, whether it can be observed at 1.5 T has not been clearly documented. The findings of existing reports are contradictory — a few earlier studies have suggested the presence of HPR in normal subjects at 1.5 T [4,5], whereas the reports by Lee and Fujii et al. do not support this conclusion [9,10]. It is thus necessary to determine whether HPR can be observed in normal subjects at 1.5 T, and, if so, to establish its MRI characteristics, and the features which distinguish it from HPR in MSA patients, at this lower signal strength.

In this study, we sought to determine whether HPR could be observed in normal subjects at 1.5 T, and to establish its prevalence, the MRI characteristics, and the distinguishing features from HPR observed in MSA patients.

## Methods

### Participants

This retrospective study was approved by the Institutional Review Board of Hokkaido University Hospital (No. 010-0017 and No. 010-0286), and complied with the ethical standards established in the Declaration of Helsinki. Written informed consent for MRI was obtained from all normal subjects. The requirement of informed consent from the patients was waived (According to the institutional regulations, written informed consent is not necessary, provided the study is retrospective and anonymity of the patients is maintained.).

To determine whether HPR could be observed in normal subjects at 1.5 T, and to establish its prevalence and MRI characteristics, the magnetic resonance (MR) images of the brains of 130 normal healthy subjects (64 men and 66 women; mean age = 43 ± 13 years; age range = 22–67 years), who volunteered to participate in establishing a normal brain database, were retrospectively reviewed. All MR images were obtained over a 6-month period (May through October, 2005). All subjects had no history of diseases which might affect the integrity of the central nervous system, such as hypertension or diabetes mellitus. Psychiatric disorders were excluded by a questionnaire. The results of neurological examination performed on the day of MRI were normal. The MR images of the brain showed no obvious abnormalities.

To establish the distinguishing features of HPR observed in MSA patients, the MR images of the brains of MSA patients obtained over a 58-month period (October, 2005 through July, 2010) were also reviewed. Inclusion criteria were probable or possible MSA according to the Consensus criteria [13] and age below 70 years. Exclusion criteria were MR images that were distorted by artifacts or MR images showing the presence of abnormalities other than those related to MSA (e.g., infarction or hemorrhage). Of the 24 MSA patients, the MR images of 6 MSA patients with predominant parkinsonian symptoms (MSA-P)(5 probable MSA-P and 1 possible MSA-P; 2 men and 4 women; mean age = 58.7 ± 4.7 years; age range = 52–64 years) and 15 MSA patients with predominant cerebellar symptoms (MSA-C)(14 probable MSA-C and 1 possible MSA-C; 7 men and 8 women; mean age = 59.2 ± 4.4 years; age range = 54–66 years) were eligible for the study. The average disease duration was 5 ± 3.7 years (range = 2–11 years) in MSA-P patients, and 4 ± 2.6 years (range = 1–9 years) in MSA-C patients.

### MRI and image processing

MRI was performed using a 1.5 T imager (Magnetom Symphony or Vision, Siemens Medical Solutions, Erlangen, Germany) and a head coil. Axial fast spin-echo T2-weighted imaging (T2WI) was performed, using the following imaging parameters: repetition time (TR) = 4500–5010 ms; echo time (TE) = 96–99 ms; effective echo train length (ETL_eff_) = 7; number of excitations (NEX) = 2; slice thickness = 5 mm; interslice gap = 1.5 mm; field of view (FOV) = 180 × 240 mm; and acquisition matrix size = 177 × 512. Axial fast spin-echo echo-planar diffusion tensor imaging (DTI) was performed in all normal subjects and 13 MSA patients (3 MSA-P, 10 MSA-C), using the following parameters: TR = 5100 ms; TE = 139 ms; b-value (b) = 1000 s mm^-2^; NEX = 2; number of gradient directions = 12; slice thickness = 5 mm; interslice gap = 1.5 mm; FOV = 240 × 240 mm; and acquisition matrix size = 92 × 128.

Mean diffusivity (MD) maps were constructed from the diffusion tensor images.

### Evaluation of images

#### Visual analysis

The axial T2-weighted images were visually evaluated by two radiologists (one with 22 years and one with 6 years of experience in neuroimaging). The evaluations were done independently, and disagreements were solved by consensus. Image evaluation was conducted in two sessions. In the first session, the T2-weighted images of normal subjects were evaluated. The raters were informed that these subjects were normal. Six months later, the T2-weighted images of all MSA patients admixed with those of 24 patients with systemic diseases other than MSA were evaluated. That is, the T2-weighted images of these 24 patients were used as dummy data, to limit potential bias; the results of these images were not used in this study. The raters were aware that the group belonged to a diseased state, but were not informed of the name of the disease or other clinical information — a situation similar to previous studies [4,14]. Evaluation was done at the foramen of Monro level, where the bilateral putamina exhibited their largest surface area. For assessment of continuity and dimension of HPR, the raters were allowed to view adjacent slices and zoom the images. However, the raters were not allowed to view images of the infratentorial compartment.

**Figure 1:**
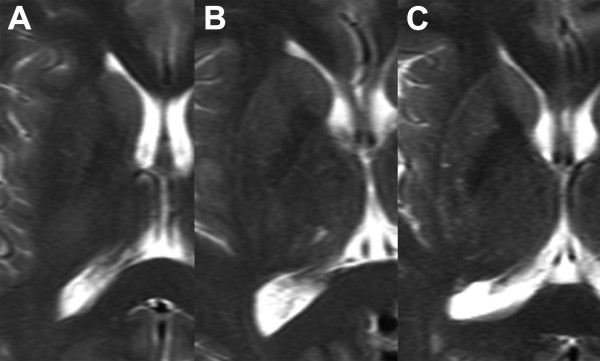
Axial T2-weighted images of normal subjects showing different degrees of conspicuity of HPR. HPR results classified as absent (A), vague (B), and present (C) are shown.

The T2-weighted images of normal subjects were evaluated for the presence of HPR, its conspicuity, distribution among gender and age groups, and the MRI characteristics {i.e., laterality, greatest dimensions (anteroposterior and transverse dimensions), association with hypointensity of the nearby putamen, and presence of discontinuity}. The conspicuity of HPR was classified into absent, vague, and present, as in Fujii et al. [10]. This classification was used so as to allow comparison of the prevalence between the two magnetic field strengths. Representative examples of the different degrees of conspicuity of HPR are shown in Figure 1. Hypointensity of the putamen was classified into mild (hyperintense to globus pallidus), moderate (isointense to globus pallidus), and severe (hypointense to globus pallidus) — depending on its signal intensity relative to globus pallidus. The MRI characteristics were determined from HPR classified as “present.”

The T2-weighted images of all MSA patients were also evaluated for the presence of HPR, its conspicuity, the MRI characteristics, and association with nearby abnormalities such as putaminal atrophy. To establish the distinguishing features of HPR, the MRI characteristics of HPR (i.e., HPR classified as “present”) were compared between the patients with MSA of the either type and age-matched normal subjects.

#### Quantitative analysis

**Figure 2:**
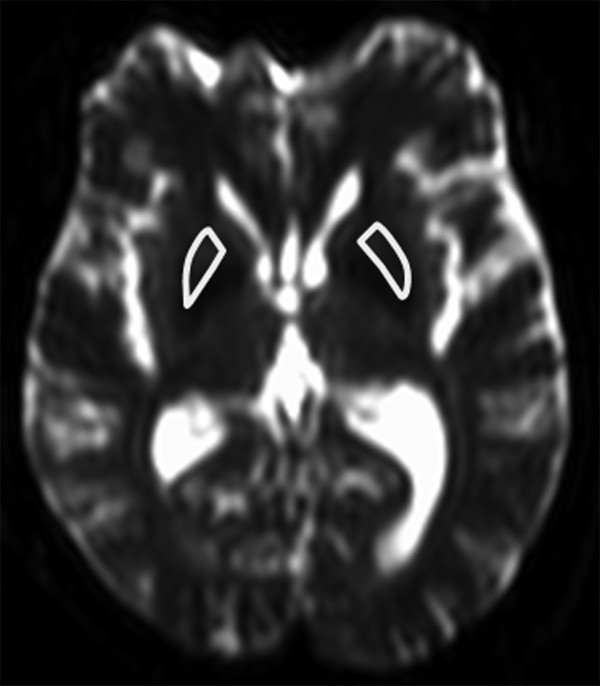
**Examples of regions-of-interest (ROIs) used in the measurement of MD values of the putamen.** The ROIs were drawn manually on the echo-planar images with no diffusion weighting, and superimposed onto the MD maps. ROIs drawn on an echo-planar image with no diffusion weighting are shown.

All measurements were performed by a radiologist with 9 years experience in neuroimaging. The MD values of the putamen were measured, using manually drawn regions-of-interest (ROIs). Measurement was performed at the foramen of Monro level. ROIs were drawn so as to include the whole putamen while avoiding inclusion of cerebrospinal fluid (CSF) or nearby structures. ROIs were drawn manually on the echo-planar images with no diffusion weighting, and superimposed directly onto the MD maps. An example of the ROIs is shown in Figure 2. The MD values of the putamen were compared between normal subjects with (i.e., HPR classified as “present”) and without HPR (i.e., HPR classified as “absent”), and between MSA patients and age-matched normal subjects with HPR.

### Statistical analysis

The MRI characteristics of HPR observed in normal subjects and the distinguishing features of HPR between normal subjects and MSA patients were established by using Fisher’s exact test. Results were considered statistically significant at p < 0.05. The MD values of the putamen were compared between normal subjects with HPR and those without HPR, by using a *t*-test. Results were considered statistically significant at p < 0.05. The MD values of the putamen were compared between MSA patients and age-matched normal subjects with HPR, by using one way analysis of variance (ANOVA) and post hoc Bonferroni tests. Results were considered statistically significant at p < 0.0167, corrected for multiple comparisons.

## Results

### Visual analysis

**Figure 3:**
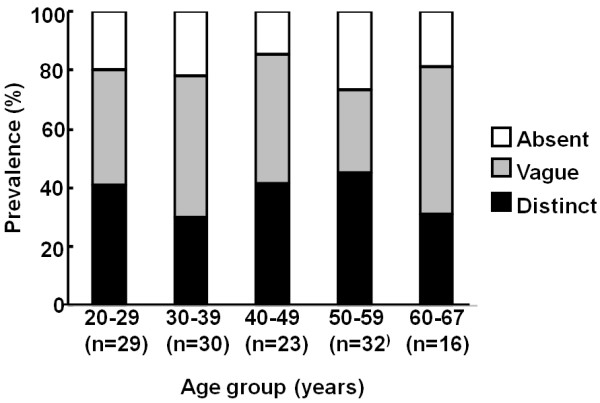
**The prevalence of HPR in normal subjects, on axial T2-weighted images.** The prevalence is given for each age group. “n” refers to the number of subjects in each age group. No significant difference in the prevalence of HPR was observed among the age groups.

Table 1 shows the overall prevalence of HPR in normal subjects. The prevalences of HPR in each age group are summarized in Figure 3. The prevalence of HPR did not vary significantly among the age groups (p = 0.49). The characteristics of HPR (HPR classified as “present”) observed in normal patients are summarized in Table 2. Neither gender predilection nor laterality in the distribution of HPR was observed. Majority of HPR were continuous throughout its length. HPR occupied nearly full or full length or the anterior aspect of the lateral margin of the putamen. All HPR measured less than 2 mm in the maximum transverse dimension. There was no significant association between the presence of HPR and hypointensity of the nearby putamen.

**Table 1:** The overall prevalence of hyperintense putamen rim (HPR) in the normal subjects on the axial T2-weighted images

**Location**	**Conspicuity of HPR**		
	**Present**	**Vague**	**Absent**
Average	38.5	40.8	20.8
Right	37.7	43.1	19.2
Left	39.2	38.5	22.3

Note: Data are presented in percentage (%).

**Table 2:** The characteristics of HPR (HPR classified as “present”) observed in normal patients

**Variable**		**Prevalence**	**P-value**♠
Gender ratio	Men	47.0%	0.33
	Women	53.0%	
Laterality	Right	49.0%	0.71
	Left	51.0%	
Maximum anteroposterior dimension	Anterior edge ~ Anterior quarter	3.0%	
	Anterior edge ~ Anterior half	37.0%	
	Anterior edge ~ Anterior three quarters	21.0%	
	Posterior edge ~ Posterior quarter	0.0%	
	Posterior edge ~ Posterior half	0.0%	
	Posterior edge ~ Posterior three quarters	0.0%	
	Nearly full to full length	39.0%	
Maximum transverse dimension	>2 mm	0.0%	
	<2 mm	100.0%	
Discontinuity of HPR	Present	21.0%	
	Absent	79.0%	
Association with hypointensity of nearby putamen	Present (Mild hypointensity)	38.0%	0.77
	Present (Moderate hypointensity)	0.6%	
	Present (Intense hypointensity)	0.0%	
	Absent	63.4%	

♠Compared with those without HPR.

**Figure 4:**
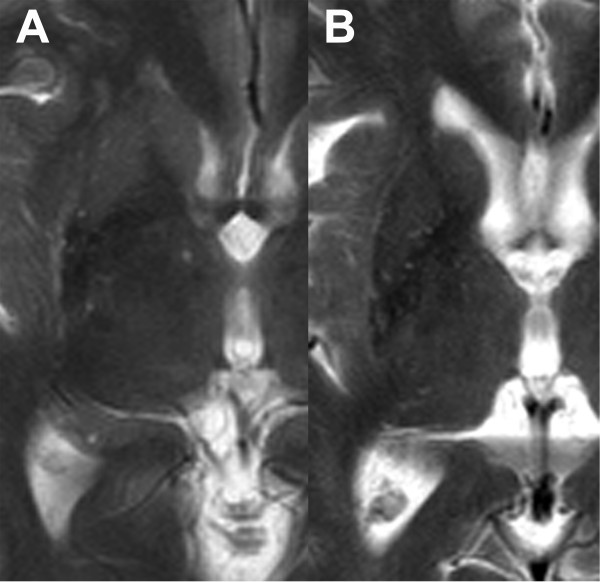
**HPR observed in MSA patients. Representative examples of HPR observed in (A) MSA-P (a 61-year-old woman; disease duration = 2 years) and (B) MSA-C (a 66-year-old woman; disease duration = 7 years) patients are shown.** In (**A**), HPR is located predominantly at the posterior part of the lateral margin of the putamen. Moderate hypointensity of the posterior half of the putamen is also seen. Putaminal atrophy is not observed in this case. HPR observed in the MSA-C patient (**B**) is not easily distinguishable from that of a normal subject (Figure 1C).

Representative examples of HPR observed in MSA-P and MSA-C patients are shown in Figure 4. HPR (i.e., HPR classified as “present”) was observed in 83.3% of MSA-P (2 men and 3 women; mean age = 57.9 ± 4.7 years; age range = 52–64 years; disease duration = 5.3 ± 3.7 years; range = 2–11 years) and 60% of MSA-C patients (4 men and 6 women; mean age = 59.1 ± 4.2 years; age range = 54–66 years; disease duration = 3.1 ± 1.8 years; range = 1–7 years). The results of the comparison of HPR among the three groups (i.e., MSA-P patients, MSA-C patients, and age-matched normal subjects) are summarized in Table 3. Compared to normal subjects and MSA-C patients, HPR was observed predominantly at a posterior location in MSA-P patients. Discontinuity of HPR and atrophy of the nearby putamen were also more frequently observed in MSA-P patients. Although not statistically significant, HPR tended to be more frequently associated with hypointensity of the nearby putamen, in MSA-P patients. The maximum transverse dimension of HPR also tended to be larger. The findings of visual analysis were similar between the normal subjects and MSA-C patients.

**Table 3:** The results of comparison of HPR (i.e., HPR classified as “present”) among MSA-P, MSA-C patients, and age-matched normal subjects

**Variables**		**MSA-P (n = 10)**	**MSA-C (n = 18)**	**Normal (n = 39)**	**P-value**
Mean age ± standard deviation (age range)		57.9 ± 4.7 years (52–64 years)	59.1 ± 4.2 years (54–66 years)	57.0 ± 3.8 years (50–66 years)	0.1883
Gender ratio	Men	40.0%	38.9%	53.9%	
	Women	60.0%	61.1%	46.1%	
Distribution of HPR	Right	60.0%	56.0%	51.0%	
	Left	40.0%	44.0%	49.0%	
Maximum anteroposterior dimension of HPR	Anterior edge ~ Anterior quarter	0.0%	5.5%	5.1%	
	Anterior edge ~ Anterior half	0.0%	16.7%	28.2%	
	Anterior edge ~ Anterior three quarters	0.0%	0.0%	23.1%	
	Posterior edge ~ Posterior quarter	0.0%	0.0%	0.0%	
	Posterior edge ~ Posterior half	40.0%	5.6%	0.0%	
	Posterior edge ~ Posterior three quarters	0.0%	0.0%	0.0%	
	Nearly full to full length	60.0%	72.2%	43.6%	
Maximum transverse dimension of HPR	>2 mm	10.0%	0.0%	0.0%	
	<2 mm	90.0%	100.0%	100.0%	
Discontinuity of HPR	Present	80.0%	38.9%	28.2%	
	Absent	20.0%	61.1%	71.8%	
Hypointensity of nearby putamen	Present	78.9%	51.6%	49.2%	
	Absent	21.1%	48.4%	50.8%	
Putaminal atrophy	Present	50.0%	11.1%	0.0%	
	Absent	50.0%	88.9%	100.0%	

Note: “n” represents the number of putamina.

* represents statistical significance.

### Quantitative analysis

**Figure 5:**
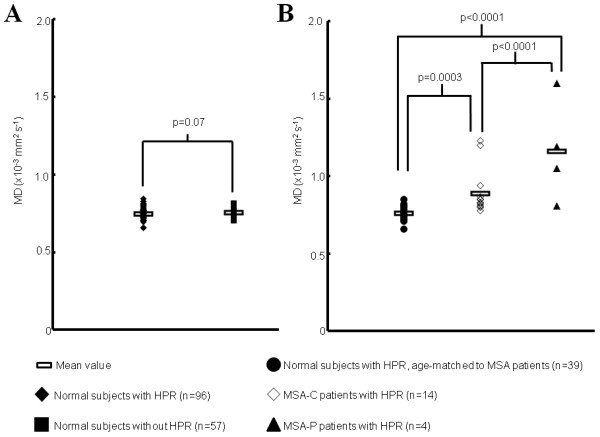
**The mean MD values of the putamen. Comparison of the MD values (A) between normal subjects with and without HPR, and (B) among normal subjects, MSA-P and MSA-C patients with HPR.** The MD values of the putamen varied significantly among the groups. Note: “n” refers to the number of putamina.

The results of quantitative analysis are summarized in Table 4 and Figure 5. There was no significant difference in the MD values of the putamen between the normal subjects with and without HPR. The mean MD values of the putamen were significantly higher in MSA-P patients than the MSA-C patients and normal subjects. Those of MSA-C patients were significantly higher than normal subjects. These differences remained statistically significant even after removal of outliers.

**Table 4:** The mean MD values of the putamen of the three groups

		**MD value(×10**^**−3**^ **s** **mm**^**−2**^**)**		**P-value**
		**Mean ± standard variation**	**Range**	
Normal subjects	with HPR	0.75 ± 0.03	0.66–0.85	0.07♠
	without HPR	0.76 ± 0.02	0.71–0.82	
MSA-P	with HPR	1.16 ± 0.33	0.81–1.60	<0.0001 (F = 15.76, df = 37)* among groups^#^; <0.0001* between MSA-P and normal or MSA-C♣; 0.0003* between MSA-C and normal♣
MSA-C	with HPR	0.89 ± 0.14	0.78–1.23	
Normal subjects (age and gender-matched to the patients)	with HPR	0.76 ± 0.04	0.66–0.85	

♠Two-sample *t*-test.

# One way ANOVA.

♣Post-hoc Bonferroni comparisons.

*represents statistical significance.

## Discussion

Our results suggest that HPR can be observed in normal subjects with a prevalence of 38.5% on T2-weighted images at 1.5 T. Compared to the report by Fujii et al. [10], our results showed a lower prevalence of HPR. This may be attributable to the difference in magnetic field strength between the two studies. The study by Fujii et al. was performed using 3 T, whereas this study was performed using 1.5 T. Imaging at 3 T allows acquisition of images with smaller pixel sizes without additional time constraints, and these images allow visualization of small or fine structures [15]. The voxel size of T2-weighted images in the study by Fujii et al. was 0.66 × 0.41 × 5 mm, whereas that in this study was 1.02 × 0.47 × 5 mm. Improved contrast between HPR and the nearby putamen can also be expected at 3 T. At 3 T, the nearby putamen is usually hypointense due to age-related ferritin and hemosiderin deposition. Hypointensity of the nearby putamen allows improved visualization of HPR. An improved contrast-to-noise ratio (CNR) may also allow improved visualization of HPR. However, a comparison of CNR between the two studies was not possible due to the insufficient information about CNR in the study by Fujii et al. It is not clear why Lee et al. failed to observe HPR at 1.5 T [9], but the limited sample size and spatial resolution along the Y-axis (i.e., frequency-encoding direction) may have played a role [9]. A difference in TE between 3 T and 1.5 T in their study as well as between their study and ours may have also been contributed.

Fujii et al. compared T2-weighted postmortem images with the histological findings in four autopsy cases who died of non-neurologic disorders, in order to explore the aetiology of HPR [10]. According to their results, HPR coincides with the myelin sparse zone at the lateral margin of the putamen. This zone is free of, or contains only scant amounts of ferritin and hemosiderin deposition. Taken together with the observation of HPR in subjects between 30 and 70 years of age (i.e. the age at which age-related iron deposition in the other parts of putamen occurs), they proposed that lack or scarcity of ferritin and hemosiderin deposition leads to hyperintensity of the zone relative to the other parts of putamen. However, in this study, HPR was observed at 1.5 T, and susceptibility effects are less pronounced at this field strength [15]. In addition, HPR was observed with equal prevalence between the younger and older age groups. There was no association between HPR and the presence of hypointensity of the nearby putamen. Failure to observe hypointensity of the putamen is consistent with the findings of a previous study [16]. It is therefore possible that the scarcity of myelin itself contributes to the appearance of HPR in normal subjects, at 1.5 T — by a mechanism similar to that responsible for hyperintensity in demyelinating and dysmyelinating lesions [17,18].

In this study, the length of HPR varied among normal subjects. The etiology underlying this variation is not known, but anatomical variation in the length of the myelin sparse zone is a possible factor. Artifacts related to CSF-flow within the third ventricle or Sylvian fissures might account for signal loss at the posterolateral aspect of putamen, but these effects are considered less likely as the images selected for use in this study were free of obvious artifacts. Although we could not determine the etiology underlying the variation in length of HPR, its predominant location can be a distinguishing feature between normal subjects and those with MSA-P. Opposed to the predominant anterior location of HPR in normal subjects, HPR was located predominantly posteriorly in MSA-P [4,19,20]. This may be due to the pathological changes of MSA (such as neuronal or axonal loss, demyelination, gliosis, tissue rarefaction, and dilatation of the perivascular spaces) which are more pronounced at the posterolateral aspect of the putamen [21]. The failure to observe the predominant posterior location of HPR in MSA-C may be due to the milder pathological changes of the putamen in MSA-C [8].

In addition to the predominant location of HPR, this study suggests some other MRI features which could help in distinguishing between normal subjects and MSA-P. In this study, discontinuity of HPR was more frequently observed in MSA-P. Discontinuity of HPR has been reported in MSA-P [11]. The reason is not known, but susceptibility effects from the nearby putamen — which has intense iron deposition — may play a role. Another feature of HPR in MSA-P is its more frequent association with putaminal atrophy [4-7,11,22]. Although not statistically significant, our results also revealed tendency toward a larger maximum transverse dimension of HPR, and an association between HPR and hypointensity of the nearby putamen, in MSA-P. The latter has also been observed in the previous reports [1-5,22]. According to these reports, hypointensity of the nearby putamen is attributable to intense deposition of iron compounds, i.e., a deposition more intense than that in the normal aging process. The MRI characteristics of HPR did not vary significantly between the MSA-C patients and normal subjects. However, MSA-C may be distinguished by abnormalities in the infratentorial compartment, such as brainstem and cerebellar atrophy, and the “hot cross bun” sign [1-8].

Quantitative analysis revealed significantly higher MD values of the putamen in MSA-P and MSA-C patients. It is considered that higher MD values are attributable to pathological changes such as neuronal loss, which destroy the tissue architecture and remove restricting barriers to water diffusion [23,24]. Alteration in MD values occurs earlier than the other MRI changes, and thus MD can be considered a potential biomarker for the early diagnosis of MSA [24,25]. Our results indicate that the MD values of the putamen may be of use in distinguishing among the three groups, in doubtful cases. Although there have been a considerable number of reports on the usefulness of MD values in distinguishing between MSA-P and PD [23,25-27], reports on the usefulness of MD values of the putamen in MSA-C are scarce. To our knowledge, there have been only two reports on the MD values of the putamen in MSA-C [27,28]. However, their results were contradictory. Ito et al. observed an equivalent degree of increase in MD values of the putamen between MSA-P and MSA-C [27], whereas Pellichia et al. did not observe any appreciable change in MD values of the putamen in MSA-C [28]. In this study, the MD values of the putamen of MSA-C patients were between those of normal subjects (The MD values of the putamen in normal subjects were comparable with those of the previous studies [26-28]) and MSA-P patients. The discrepancy may be at least partly related to the limited number of patients enrolled in these studies and the variations in disease duration or severity. Further studies will be needed to explore this issue.

This study has a few limitations. First, we did not include normal subjects who were below 22 or above 67 years of age. Therefore, we were unable to examine whether HPR appears in younger individuals or whether it becomes indistinct in older individuals, as reported by Fujii et al. [10]. Second, the number of MSA-P patients was limited. This may have been attributable to the fact that MSA-C is the predominant clinical phenotype in Japan [29]. Third, the present study did not include patients with PD, progressive supranuclear palsy, and corticobasal degeneration. Some studies have reported that HPR can also be observed in these patients [4,14,22]. Because the present study did not include these patients, we could not determine whether the HPR observed in normal subjects was different from that observed in these diseases. However, according to the findings of previous reports which revealed HPR in these diseases, HPR may not be distinguishable between these diseases and normal subjects, and HPR may be a normal finding in these diseases. Finally, in this study, a rough comparison of the prevalence of HPR among the studies was made. Ideally, identical scan parameters are desired. As variation in the scan parameters can lead to variation in the conspicuity of HPR, quantification of T2 values, rather than evaluation of signal intensity on T2-weighted images, might be better suited for future studies.

## Conclusions

This study documented the appearance of HPR in normal subjects at 1.5 T. HPR may be observed in 38.5% of normal subjects on T2-weighted images at 1.5 T. Thin linear hyperintensity, which occupies the full length or anterior half of the lateral margin of the putamen and shows continuity throughout its length, is suggestive of “normal.” In doubtful cases, measurement of MD values of the nearby putamen may be valuable in distinguishing among normal, MSA-P, and MSA-C patients. Radiologists and neurologists should be aware that HPR may be observed in normal subjects at 1.5 T— so as to avoid misinterpretation of normal HPR as a sign of pathological state.

## Competing interests

The authors declare that they have no competing interests.

## Authors’ contributions

KKT and ST are the guarantors of the integrity of the entire study. KKT contributed to the study design, preparation for visual analysis, quantitative analysis, statistical analysis, literature research, and writing of the manuscript. ST contributed to the study design, visual analysis, and manuscript editing. AT contributed in visual analysis and manuscript editing. RM contributed in preparation for visual analysis and literature research. H. Soma, IY, and H. Sasaki contributed in clinical studies and manuscript editing. YMI contributed in statistical analysis and manuscript editing. H. Shirato contributed in overall management and manuscript editing. All authors read and approved the final manuscript.

## Pre-publication history

The pre-publication history for this paper can be accessed here:

http://www.biomedcentral.com/1471-2377/12/39/prepub
